# Supplementary material for: Prospects of and Barriers to the Development of Epitope-Based Vaccines against Human Metapneumovirus
Source: Pathogens. 2020 Jun 18;9(6):481. doi: 10.3390/pathogens9060481 (PMC7350342; doi:10.3390/pathogens9060481)
Supplement: Supplementary file 1 [file pathogens-09-00481-s001.pdf]

Supplementary material to the manuscript "Prospects of and Barriers to the Development of Epitope-Based Vaccines against Human Metapneumovirus"

Table S1. Human metapneumovirus epitopes with confirmed natural processing, deposited in IEDB

IEDB ID	Epitope sequence	Protein (position)	Allele	Model	Assay				Ref.
					ELISPOT/ICS IFNγ release	51 chromium cytotoxicity	Pathogen burden after challenge	Degranulation	
547003	NGVRVLATA*	F (153-161)	HLA-A*02:01	PBMC		+			[1]
539306	GIQYISTAL	N (66-74)	HLA-A*02:01	Tg mice	+				[2]
539477	KKFEVNATV	M (86-94)	HLA-A*02:01	Tg mice	+				[2]
539532	KVYYRSLFI	N (217-225)	HLA-A*02:01	Tg mice	+				[2]
539533	KVYYRSLFIEYGKAL	N (217-231)	HLA-A*02:01	Tg mice	+				[2]
539776	PKKFEVNATVALDEY	M (85-99)	HLA-A*02:01	Tg mice	+				[2]
540147	YAAEIGIQYISTALG	N (61-75)	HLA-A*02:01	Tg mice	+				[2]
540151	YKHAILKESQYTIKR	N (13-27)	HLA-A*02:01	Tg mice	+				[2]
159213	YAAEIGIQY	N (61-69)	HLA-A*02:01	Tg mice	+				[2]
539268	FQANTPPAV*	M (39-47)	HLA-A*02:01	PBMC/Tg mice	+		+		[2]
159112	SLQQEITLL*	N (39-47)	HLA-A*02:01	PBMC/Tg mice	+				[2]
69387	VLATAVREL*	F (157-165)	HLA-A*02:01	PBMC	+	+			[3]
31915	KLILALLTFL	SH (35-44)	HLA-A*02:01	Tg mice	+	+			[4,5]
59182	SLILIGITTL	G (32-41)	HLA-A*02:01	PBMC/Tg mice	+	+			[3,5]
31756	KLAKLIIDL	M2-1 (157-165)	HLA-A*02:01	PBMC	+	+			[3]
25388	IAPYAGLIMI*	M (194-203)	HLA class I	PBMC	+	+			[3]
33979	KVEGEQHVIK*	F (429-438)	HLA class I	PBMC	+	+			[3]
54706	RLPREKLKK	M2-1 (149-157)	HLA-A*11	PBMC	+	+			[3]
28126	IPYTAAVQV*	M (12-20)	HLA-B*07	PBMC	+	+			[3]
32663	KPAVGVYHIV	SH (152-161)	HLA-B*07	PBMC	+	+			[3]
158691	APYAGLIMI*	M (195-203)	HLA-B*07:02	Tg mice	+			+	[6,7]
159244	YPRMDIPKI	N (198-206)	HLA-B*07:02	Tg mice			+		[6]
60092	SPKAGLLSL*	N (307-315)	HLA-B*07	PBMC	+	+			[3]
			H2-Ld	BALB/c mice	+	+	+		[8]
68624	VGALIFTKL	N (164-172)	HLA-A*02:01	Tg mice	+	+			[5]
			H2-Kb	C57bl/6 mice	+	+			[4,5]
178408	SGVTNNGFI	F (528-536)	H2-Db	C57bl/6 mice	+			+	[6,7,9,10]
178390	LSYKHAIL	N (11-18)	H2-Kb	C57bl/6 mice	+			+	[6,7,10]
7407	CYLENIEII	M2-2 (56-64)	H2-Kd	BALB/c mice	+	+			[5,8]
5782	AYGAGQTML	N (251-259)	H2-Kd	BALB/c mice	+				[8]
6813	CPNFASVVL	N (318-326)	H2-Ld	BALB/c mice	+				[8]
7393	CYKGVSCSI	F (384-392)	H2-Kd	BALB/c mice	+				[8]
21782	GPSLIKTEL	F (63-71)	H2-Ld	BALB/c mice	+				[8]
23359	GYIDDNQSI	M2-1 (81-89)	H2-Kd	BALB/c mice	+	+			[8]
29615	IYLIINYTI	G (47-55)	H2-Kd	BALB/c mice	+				[8]
30879	KFVSSAKPV	M (128-136)	H2-Kd	BALB/c mice	+				[8]

38414	LPASLTIWF	M 928-36)	H2-Ld	BALB/c mice	+				[8]
45470	NPRQSRFVL	F (97-105)	H2-Ld	BALB/c mice	+				[8]
65510	TPASLINNL	M (139-147)	H2-Ld	BALB/c mice	+				[8]

* epitopes located within conserved protein region

Supplementary references:

1. Szomolay, B.; Liu, J.; Brown, P.E.; Miles, J.J.; Clement, M.; Llewellyn-Lacey, S.; Dolton, G.; Ekeruche-Makinde, J.; Lissina, A.; Schauenburg, A.J.; et al. Identification of human viral protein-derived ligands recognized by individual MHCI-restricted T-cell receptors. Immunol. Cell Biol. 2016, 94, 573–582, doi:10.1038/icb.2016.12.
2. Hastings, A.K.; Gilchuk, P.; Joyce, S.; Williams, J.V. Novel HLA-A2-restricted human metapneumovirus epitopes reduce viral titers in mice and are recognized by human T cells. Vaccine 2016, 34, 2663–2670, doi:10.1016/j.vaccine.2016.04.034.
3. Herd, K.A.; Nissen, M.D.; Hopkins, P.M.; Sloots, T.P.; Tindle, R.W. Major histocompatibility complex class I cytotoxic T lymphocyte immunity to human metapneumovirus (hMPV) in individuals with previous hMPV infection and respiratory disease. J. Infect. Dis. 2008, 197, 584–592, doi:10.1086/526536.
4. Chen, D.; Edgtton, K.; Gould, A.; Guo, H.; Mather, M.; Haigh, O.; Cochrane, M.; Kattenbelt, J.; Thomson, S.; Tindle, R. HBsAg-vectored vaccines simultaneously deliver CTL responses to protective epitopes from multiple viral pathogens. Virology 2010, 398, 68–78, doi:10.1016/j.virol.2009.11.042.
5. Herd, K.A.; Mahalingam, S.; Mackay, I.M.; Nissen, M.; Sloots, T.P.; Tindle, R.W. Cytotoxic T-lymphocyte epitope vaccination protects against human metapneumovirus infection and disease in mice. J. Virol. 2006, 80, 2034–2044, doi:10.1128/JVI.80.4.2034-2044.2006.
6. Erickson, J.J.; Gilchuk, P.; Hastings, A.K.; Tollefson, S.J.; Johnson, M.; Downing, M.B.; Boyd, K.L.; Johnson, J.E.; Kim, A.S.; Joyce, S.; et al. Viral acute lower respiratory infections impair CD8+ T cells through PD-1. J. Clin. Invest. 2012, 122, 2967–2982, doi:10.1172/JCI62860.
7. Erickson, J.J.; Lu, P.; Wen, S.; Hastings, A.K.; Gilchuk, P.; Joyce, S.; Shyr, Y.; Williams, J.V. Acute Viral Respiratory Infection Rapidly Induces a CD8+ T Cell Exhaustion-like Phenotype. J. Immunol. Baltim. Md 1950 2015, 195, 4319–4330, doi:10.4049/jimmunol.1403004.
8. Melendi, G.A.; Zavala, F.; Buchholz, U.J.; Boivin, G.; Collins, P.L.; Kleeberger, S.R.; Polack, F.P. Mapping and characterization of the primary and anamnestic H-2(d)-restricted cytotoxic T-lymphocyte response in mice against human metapneumovirus. J. Virol. 2007, 81, 11461–11467, doi:10.1128/JVI.02423-06.
9. Cox, R.G.; Erickson, J.J.; Hastings, A.K.; Becker, J.C.; Johnson, M.; Craven, R.E.; Tollefson, S.J.; Boyd, K.L.; Williams, J.V. Human metapneumovirus virus-like particles induce protective B and T cell responses in a mouse model. J. Virol. 2014, 88, 6368–6379, doi:10.1128/JVI.00332-14.
10. Erickson, J.J.; Rogers, M.C.; Hastings, A.K.; Tollefson, S.J.; Williams, J.V. Programmed death-1 impairs secondary effector lung CD8+ T cells during respiratory virus reinfection. J. Immunol. Baltim. Md 1950 2014, 193, 5108–5117, doi:10.4049/jimmunol.1302208.
